# Developing leadership skills by participating in a peer-led committee of junior doctors

**DOI:** 10.15694/mep.2021.000079.1

**Published:** 2021-03-26

**Authors:** Layla Hehir, Jenny Dodds, Helen Rooney, Megan Anakin

**Affiliations:** 1 Southern District Health Board; 2 University of Otago

**Keywords:** leadership, engagement, junior doctors, committee, experiential learning, community of practice, medical education

## Abstract

This article was migrated. The article was marked as recommended.

**Background.** This article uses a case study approach to describe and analyse how a peer-led committee was used to develop leadership skills among junior doctors. Junior doctors are a potentially powerful group of leaders within the healthcare sector, yet more senior staff members may encounter difficulty engaging with this group. Leadership and engagement are essential for optimal functioning of an organisation.

**Alternatives.** Typical methods used to develop leadership skills and promote engagement with leadership activities include higher degrees, short courses, coaching, and experiential learning activities. One alternative can be to use experiential learning that has be informed by situated learning theory and the concept of communities of practice.

**Solution.** A peer-led committee of junior doctors was established using to develop leadership skills and promote engagement with leadership activities. The committee was designed to address five features of a community of practice: mutual engagement, joint enterprise, shared repertoire, learning, and community. This peer-led committee included elected roles and met regularly out of hours over two sites within the Southern District Health Board, New Zealand.

**Recommendations.** This intervention was well-received by junior doctors and has been sustained in the Southern District Health Board. A peer-led committee structured with the features of a community of practice may be useful method for others to use when they seek to promote and support the development leadership skills of junior doctors.

## Background

There is increasing awareness of the need for doctors to be effective leaders ( Clark, Spurgeon and Hamilton, 2008). There has been criticism that doctors, at all levels of practice, do not demonstrate the expected level of leadership skills to optimise effective delivery of healthcare ( Dowton, 2004). Healthcare leadership has been described as ‘the behaviour of an individual when directing the activities of a group toward a shared goal’ ( Al-Sawai, 2013) and is instrumental in achieving good organisational outcomes ( Sfantou *et al.,* 2017). Medical leadership can include resource managing, decision making, recruiting and medical consulting, as well as implementing changes and improvements in hospital and clinical settings ( Chadi, 2009). Leadership is a part of clinical practice even for those who never undertake a formal leadership role. In New Zealand, leadership is considered a core competency by the Royal Australasian College of Physicians (2020) and Royal Australasian College of Surgeons (2020).

Medical engagement has also been found to be key in effective running of a healthcare organisation (Markos and Sridevi, 2010). Employee engagement has been defined as “a positive attitude held by the employee towards the organization and its value” ( Rimmer, 2016), and is closely related to, though distinct from, job satisfaction. Effective leadership has been found to be correlated with increased workplace engagement, and both have been associated with increased workplace success ( Vincent‐Höper, Muser and Janneck, 2012).

Junior doctors are important group of potential leaders in the healthcare field, yet more senior staff report difficulty to engage with this group ( Rimmer, 2016). The term junior doctors in this paper refers to doctors in the first few years of medical practice and includes resident medical officers who are undertaking more specialised medical training. Although training bodies consider leadership to be a core competency, there may be a lack of opportunity to develop leadership skills for junior doctors not yet associated with a specific college, with junior doctors viewed as transient employees rather than potential leaders ( Till, 2015). Worldwide, common methods to improve leadership skills include courses, higher degrees, coaching, and experiential learning activities ( Warren and Carnall, 2011). Each of these methods will be explored as possible options to provide junior doctors with leadership skills and promote engagement with leadership activities in their workplaces.

## Alternatives

Leadership can be taught using short courses, either in person or online. Examples of courses in leadership in the UK include the Oxford Course in Leadership and management in Healthcare ( University of Oxford, 2020) and the online course by ISC Learn (2020). In New Zealand, there are courses available to develop leadership skills for medical personnel such as the summer course offered by the University of Otago (2020). Advantages of these courses are that junior doctors can attend while being employed full time, they receive formal leadership training, and they have the opportunity to network with like-minded people in different institutions. Disadvantages of short courses include need for junior doctors to obtain funding to pay for each course and a lack of tailored courses specific for junior doctors. In addition, not all junior doctors may be interested in attending a course or able to secure a place.

Another method used to develop leadership skills is for junior doctors to undertake a higher degree such the postgraduate degree in healthcare leadership. Examples of healthcare leadership degrees include in-person and distance study options at universities in the USA (e.g., Harvard Medical School, 2020), the UK (e.g., Royal College of Physicians, 2020), and New Zealand (e.g., University of Auckland, 2020). Advantages of undertaking a higher degree include developing a highly trained workforce with specific leadership expertise and for individuals to obtain a formal qualification. Disadvantages of this method are that only a limited number of doctors may be able to pursue and complete a higher degree as a formal leadership qualification. With short courses and higher degrees, there is a risk that the skills learned may not translate to improved leadership behaviours in the clinical setting.

Coaching is a form of teaching frequently used to develop leadership skills ( Garvey, Strokes and Megginson, 2010). Coaching involves a relationship between two or more individuals based around defined goals, usually with a specific timeframe in mind. This has been used specifically to develop leadership skills within healthcare ( Fielden, Davidson and Sutherland, 2009) Advantages of coaching include the opportunity to develop a lasting collegial relationship and the potential to develop specific personal goals with one’s coach. Disadvantages may include difficulty engaging, matching coaching styles to individuals and maintaining the relationship within a working environment with frequent staff rotation.

Experiential learning activities can occur in clinical workplaces and allow junior doctors to reflect on experiences they have had and transform them into knowledge that can be applied in other contexts ( Kolb, 2015). Advantages of experiential learning activities are that there may be no external time pressure or funding required and that they can be experienced by a large group of junior doctors. Disadvantages are that experiential learning activities can be opportunistic and idiosyncratic, and therefore difficult to standardise. They may also require a work environment that is conducive to learning.

Situated learning theory ( Lave and Wenger, 1991) can provide a theoretical resource that can be to enhance and fortify experiential learning activities. For example, the concept of a community of practice can be used to understand how a group of individuals may engage in a process of collective learning in a shared domain of human endeavour ( Hara, 2009; Wenger, McDermott and Snyder, 2002). There are five key features in a community of practice: mutual engagement, joint enterprise, shared repertoire , learning, and community ( Mercieca, 2017). Advantages of considering the features of a community of practice when designing and implementing an experiential learning activity include being aware of the benefits of collaborating with a like-minded group and adding structure to experiential learning. Disadvantages may include difficulties when managing the group’s expectations and the time commitment required in order to organise and maintain the group.

## Solution

We made the decision to establish a peer-led committee to develop leadership skills and increase engagement among junior doctors. This decision involved including features of a community of practice to allow junior doctors to learn from each other in an informal setting. Peer-led teaching and learning has been found to help to create a positive learning environment ( Ologunde and Rabiu, 2014), therefore, the group was designed to allow junior doctors to engage with each other and learn from one another by working towards shared goals. The variety of activities promoted by the peer-led committee were aimed at reaching a diverse group of junior doctors by creating a fun and enjoyable learning environment. The activities were also designed to help translate leadership experiences to the workplace in the absence of external funding. Consequently, the peer-led committee was established at two sites in 2017 in Southern District Health Board, New Zealand, with the goal of delivering identified interventions to improve junior doctor engagement and offer an opportunity to develop leadership skills. The aims of the peer-led committee included building collaboration, providing more opportunities to engage in leadership activities and improving the relationship between junior doctors and District Health Board leadership. The peer-led committee developed a vision and mission statement, under the guidance of a quality and performance facilitator which included formal roles that are filled by an annual election (see Table 1).

**Table 1:** Vision and Mission Statements and the Roles of the Peer-led Committee

**VISION** Our vision is for all junior doctors to work in an environment where they feel valued and inspired with access to opportunities to develop and reach their full potential; empowering and enabling them to be the best doctors they can be.
**MISSION** Our mission is to maintain a sustainable and adaptable committee. The committee will provide, facilitate and publicise opportunities for junior doctors, both professional and social, and will foster dialogue with DHB leadership, achieving tangible and accountable outcomes. We will achieve our mission by having transparent processes, regular meetings and providing continuous and varied opportunities for feedback from junior doctors. As a group we are committed to being inclusive, accountable, visible and fun. Roles are elected by the junior doctors and new roles have been created based on feedback.
**Roles** President Secretary Treasurer Welfare Representative Arts and Culture Representative Sustainability Representative Sports Representative Communications Representative Education and Professional Development Representative Social Representative Union Representative

Since its establishment, the peer-led committee continues to meet on a fortnightly to monthly basis, with regular communication between sites. The peer-led committee supports regular social events as a means of encouraging strong social relationships between junior doctors with the understanding that these relationships have a beneficial psychosocial role in their overall wellbeing. The peer-led committee runs a mentorship programme, interdepartmental quiz night, a yearly ski trip and an annual hospital ball.

The peer-led committee support a monthly forum where junior doctors have an opportunity to raise concerns and discuss current issues in a safe space. This forum is chaired by committee members and all local junior doctors are invited to attend. Issues that require escalation are raised to a quarterly governance group which is attended by senior management of the hospital. The peer-led committee communicates by a monthly email update, Facebook, Instagram, and a quarterly newsletter. Feedback from participating junior doctors is encouraging. The majority of junior doctors who participate in the peer-led committee’s activities report an interest in leadership and appreciate the opportunity to meet like-minded individuals. Challenges reported centred around two main themes; time commitment and difficulty engaging their peers. To address these challenges, committee members may wish to a measuring pre- and post-participation leadership behaviours and attitudes using survey, interviews or focus group methods.

## Recommendations

Others interested in supporting leadership development among their junior doctors may find that drawing on the features of a community of practice might be beneficial. The peer-led committee appears to be a sustainable intervention. By establishing fixed roles that are renewed regularly, the committee is given stability despite the transient nature of the junior doctor workforce ( Rimmer, 2016). By engaging with hospital management, the committee has helped to establish relationships between junior doctors and management and provided a way to contact junior doctors in other sites. The peer-led committee also affords autonomy to its participants. To address issues that might arise around expectations of time commitments and understanding of the responsibilities of each role, other groups may wish to consider developing an agreed memorandum of understanding around expected roles and responsibilities of participants.

The peer-led committee model may be useful for other junior doctors and managers in New Zealand to use when seeking to develop leadership skills among junior doctors. Next steps might be to scale up this model to form a national committee, as has been done in the UK ( British Medical Association, 2020). In New Zealand, a peer-led committee appears to be a useful and sustainable way to promote engagement and develop leadership skills among junior doctors. As this is a notoriously difficult group to engage ( Rimmer, 2016), using a junior doctor-led committee designed with the features of a community of practice may be implemented in other District Health Boards, institutions, and with other groups of professionals.

## Take Home Messages

• Both engagement and leadership are important to achieve good organisational outcomes.
• Junior doctors are potentially powerful leaders within the healthcare system but engagement of this group can be challenging.
• A peer-led committee may be useful intervention for developing leadership skills and increasing engagement among junior doctors.
• The functioning peer-led committee can be informed by Situated Learning Theory and the features of a community of practice.
• Others may find this model useful for developing leadership skills among junior doctors in their healthcare context.

## Notes On Contributors

Layla Hehir MbBchBAO National University of Ireland, Galway. Southern District Health Board, Dunedin, New Zealand. ORCID ID: https://orcid.org/0000-0002-2201-8522

Jenny Dodds MBChB BSc Hons University of Bristol. Southern District Health Board, Dunedin, New Zealand.

Helen Rooney Registrar MBChB BEng University of Manchester. Southern District Health Board, Dunedin, New Zealand.

Dr Megan Anakin, BSc (McGill), BFA (NSCAD), BEd (Dalhousie), MEd (Simon Fraser), PhD (Otago), AFANZAHPE, Lecturer and Education Adviser, Education Unit, Otago Medical School - Dunedin Campus, University of Otago, Dunedin, New Zealand. ORCID ID: https://orcid.org/0000-0002-6499-7802

## Declarations

For transparency, Megan Anakin is a member of the Editorial Board of MedEdPublish.
