# Supplementary material for: Developing leadership skills by participating in a peer-led committee of junior doctors
Source: MedEdPublish (2016). 2021 Mar 26;10:79. [Version 1] doi: 10.15694/mep.2021.000079.1 (PMC10939646; doi:10.15694/mep.2021.000079.1)
Supplement: Peer review history

# Reviewer report: Reviewer response for version 1

**DOI:** 10.21956/mep.18958.r27136

**Published:** 2021-06-14

**Authors:** Ken Masters

**Affiliations:** 1 Sultan Qaboos University

This review has been migrated. The reviewer awarded 3 stars out of 5 An interesting novel idea to developing leadership skills by participating in a peer-led committee of junior doctors.The paper establishes the need for leadership training among doctors, and then explores some of the methods currently used for such training, including short courses, a full degree, coaching, experiential learning, and situated learning.It then discusses the approach followed.Some issues do need to be addressed:• A little more detail on the setting is required. This should come after the “Alternatives” and could probably include some material currently in the “Solution” section. This is necessary so that the reader can have a clearer understanding of the context in which the solution was implemented.• This setting should also explain why the possible solutions outlined above were not suitable. (The disadvantages of each system are given, but one would need to understand the setting, and the relative importance of those disadvantages to the setting in order to understand why they were not chosen).• It’s probably not necessary to repeat “peer-led” in the mention of the Committee.• I think a little more information on the activities is required. In particular, it would be useful to know numbers of people attending the various events, on the mailing lists, etc. Some sort of impact measurement is required.• Relating back to the points above, it would also be useful if the paper could give some idea of the benefits of using this approach rather than the other methods outlined (e.g. overall achievements that could not have been achieved through the other methods, or cheaper, more effective, etc.). In the absence of any kind of evaluation (which could easily be done with courses, for example), this is necessary for the reader to gain an overall feel for the success of the process. So, I think that the authors have found a system that works for them, but there is quite a bit of information missing that the reader would need before having an appreciation of what has been achieved. I look forward to Version 2 of the paper in which these issues are addressed.

Reviewer Expertise:

NA

No decision status is available

# Reviewer report: Reviewer response for version 1

**DOI:** 10.21956/mep.18958.r27137

**Published:** 2021-05-17

**Authors:** Madalena Folque Patricio

**Affiliations:** 1 School of Medicine, University of Lisbon

This review has been migrated. The reviewer awarded 4 stars out of 5 Thanks for this important paper entitled ‘Developing leadership skills by participating in a peer-led committee of junior doctors’.The authors report not only a growing awareness of the need for doctors to be effective leaders but also on the recognition of leadership as a core competency by the Royal Australasian College of Physicians (2020) and Royal Australasian College of Surgeons (2020). To reinforce the above, I would like to draw attention to the 2010 Lancet Report (The Lancet, Vol. 376, No. 9756, p1877–1878), which, while advancing third-generation reforms, the Commission presents a vision for transformative learning, as the highest of three successive levels: Informative learning is about acquiring knowledge and skills, to produce experts. Formative learning is about socializing students around values to produce professionals. Transformative learning is about developing leadership attributes to produce enlightened change agents in society. This is extremely important in a moment when medical schools are under an enormous pressure from social accountability. Authors should be congratulated for • Drawing attention to the importance of training doctors as future leaders namely writing a good Background to introduce the problem and its context • Giving a good description of pre-existing alternative models for training leaders• Proposing a new model based on a peer-led committee to develop leadership skills and increase engagement among junior doctors • Describing the main steps on the new modelThat said, I would like to suggest to the authors that they give more details on the new model (for example, by adding some appendices to clarify the different steps) because I doubt that other schools would be able to replicate the model based on what has been described .In addition, if it was good to hear that the majority of junior doctors who participate in the peer-led committee’s activities report an interest in leadership and appreciate the opportunity to meet people with similar ideas and that major challenges were around time commitment and difficulty to engage their peers, I would expect to have found more detailed evaluation results, namely the number of participants and response rate for each question, main strengths as well as main difficulties in addition to the two above themes. A small point is that the use of the DHB abbreviation for ‘District health boards’ is not justified because it only appears once. If introduced it should first have to be spelled out in full, since it is the first time it appears in the text. Finally let me conclude by saying that this study would be useful to medical schools who wish to implement transformative teaching namely to develop students’ leadership skills as a core competency of the future Doctor.

Reviewer Expertise:

NA

No decision status is available
